# Teledermatology Consults in a County Hospital Setting: Retrospective Analysis

**DOI:** 10.2196/30530

**Published:** 2021-09-08

**Authors:** Colton H Funkhouser, Martha E Funkhouser, Jay E Wolverton, Toby Maurer

**Affiliations:** 1 Georgetown University School of Medicine Washington, DC United States; 2 Department of Dermatology San Mateo Medical Center San Mateo, CA United States; 3 Department of Dermatology Indiana University School of Medicine Indianapolis, IN United States

**Keywords:** dermatology, teledermatology, telemedicine, referrals, primary care, keratosis, digital health, skin cancer, dermatitis

Teledermatology is increasingly used by primary care providers (PCPs) for diagnosis and triage of skin conditions [1,2]. Many dermatology practices have increased telemedicine services in light of the COVID-19 pandemic [2]. Current teledermatology guidelines provide standards for effective teledermatology practice but do not detail recommendations for management of specific conditions [2]. By understanding the distribution of cases sent to teledermatology, and which are seen in-person, guidelines can be properly structured to optimize teledermatology use.

Prior studies have found that 20% to 50% of teledermatology cases required an in-person visit after teledermatology evaluation [3-5]. However, there is limited information on the distribution of cases sent for teledermatology consultation. In our study, teledermatology consults from PCPs at a county hospital were analyzed to identify common diagnoses that prompted the use of the teledermatology system and which diagnoses required an in-person visit. PCPs were encouraged to send any dermatologic cases to teledermatology, even if they felt comfortable managing it independently.

We conducted a retrospective analysis of 450 store-and-forward consults from PCPs to teledermatologists via Medweb from 2017 to 2019 at San Mateo County Medical Center in California. Diagnoses were made by the teledermatologist based on the teledermatology consult. Our analysis captured 471 diagnoses encompassing a wide range of dermatologic conditions (Table 1). The most frequent diagnoses were seborrheic keratosis, eczema, and acne. Overall, 39.9% of diagnoses seen via teledermatology were referred for an in-person visit, the most common of which were nonmelanoma skin cancer, actinic keratosis, and alopecia areata. Others such as atopic dermatitis and lentigo were never referred for an in-person visit. When grouped into categories based on similar types of dermatologic diseases (Figure 1), the most frequent group was banal and precancerous neoplasms. The groups with the highest proportion of referrals for in-person visits were malignant neoplasms and hair disorders. The papulosquamous disorders and acneiform disorders groups were referred for an in-person visit less frequently. We found that 6.2% of consults could not be diagnosed via teledermatology due to insufficient photo quality or patient history.

**Table 1:** Top 25 diagnoses sent to teledermatology listed in order of frequency and the proportion requiring referral to an in-person visit.

Diagnosis	Cases, n	Referred, n (%)	Not referred, n
Seborrheic keratosis	48	4 (8)	44
Eczema NOS^a^	30	1 (3)	29
Acne	27	6 (22)	21
Rule out NMSC^b,c^	28	28 (100)	0
Seborrheic dermatitis	20	2 (10)	18
Actinic keratosis	17	17 (100)	0
Poor photo quality	12	8 (67)	4
Vitiligo	12	4 (33)	8
Banal neoplasm NOS	12	9 (75)	3
Insufficient data	11	7 (64)	4
Wart	11	10 (91)	1
Nevus	10	6 (60)	4
Contact dermatitis	9	3 (33)	6
Alopecia areata	8	8 (100)	0
Rosacea	8	2 (25)	6
Papulosquamous disorder NOS	8	2 (25)	6
Cyst	8	3 (38)	5
Keloid	6	5 (83)	1
Dermatologist unable to make diagnosis	6	4 (67)	2
Onychodystrophy NOS	6	2 (33)	4
Atopic dermatitis	6	0 (0)	6
Lentigo	6	0 (0)	6
Idiopathic guttate hypomelanosis	5	2 (40)	3
Urticaria	5	1 (20)	4
Angioma	5	3 (60)	2

^a^NOS: not otherwise specified.

^b^NMSC: nonmelanoma skin cancer.

^c^NMSC includes basal cell carcinoma, squamous cell carcinoma, and dermatofibroma sarcoma protuberans.

**Figure 1:**
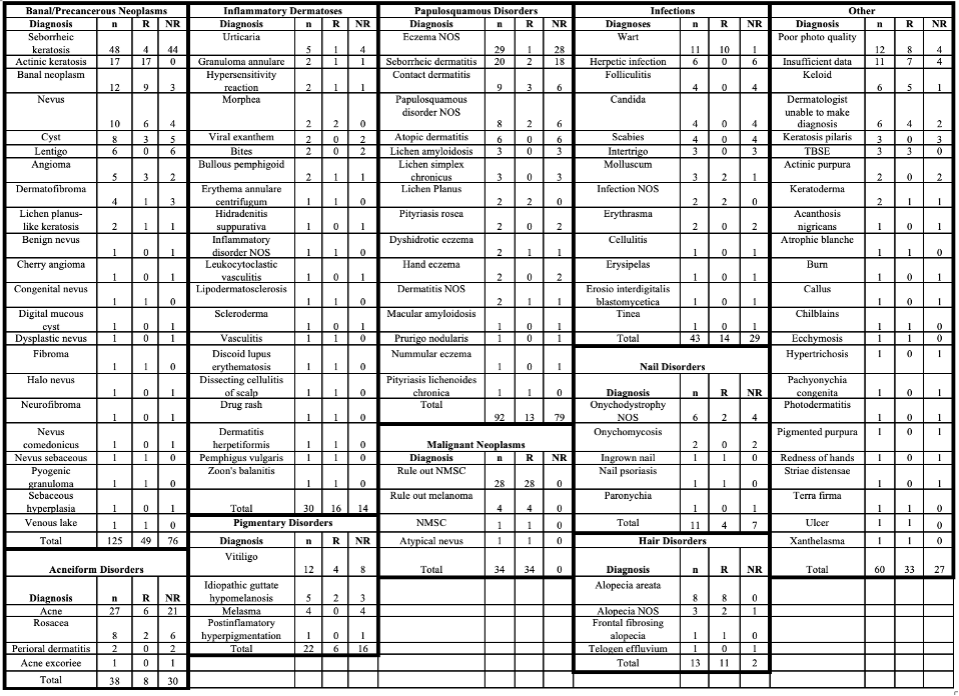
Diagnoses referred to teledermatology grouped into categories based on similarity. TBSE was due to: patient high risk, patient history of melanoma/NMSC, and patient request. NMSC: nonmelanoma skin cancer; NOS: not otherwise specified; NR: not referred; R: referral; TBSE: total body skin exam.

Our study demonstrates that teledermatology is frequently used to manage benign skin conditions while serving as a triage tool for more concerning lesions that should be evaluated in person. The diagnoses most commonly referred for an in-person visit were ones with concern for precancer or malignancy, or that required procedural management, such as alopecia areata, verruca, and keloids. Furthermore, hair disorders and scalp lesions can be difficult to capture via photo and frequently necessitated an in-person visit. Benign conditions without concern for malignancy were able to be managed completely via teledermatology.

The results of this study can provide support for guidelines delineating which dermatologic conditions are appropriate to be managed via teledermatology and which require in-person management. There are several limitations of this study: it did not specifically quantify the severity of disease, it did not follow long-term outcomes of cases managed via teledermatology, and it focused on patients only in a county hospital setting. Future work should focus on addressing these limitations with studies in other patient populations to provide more robust support for teledermatology guidelines.

## Abbreviations

PCP: primary care provider
